# Impact of volatility reduction agents on dicamba and glyphosate spray solution pH, droplet dynamics, and weed control

**DOI:** 10.1002/ps.7258

**Published:** 2022-11-22

**Authors:** Koffi Badou‐Jeremie Kouame, Thomas R. Butts, Rodrigo Werle, William G. Johnson

**Affiliations:** ^1^ Postdoctoral Research Fellow, Department of Crop, Soil, and Environmental Sciences University of Arkansas System Division of Agriculture Lonoke AR USA; ^2^ Extension Weed Scientist, Department of Crop, Soil, and Environmental Sciences University of Arkansas System Division of Agriculture Lonoke AR USA; ^3^ Extension Weed Scientist, Department of Agronomy University of Wisconsin‐Madison Madison WI USA; ^4^ Weed Scientist, Department of Botany & Plant Pathology Purdue University West Lafayette IN USA

**Keywords:** tank‐mixture, VRA, antagonism, droplet size, droplet velocity

## Abstract

**BACKGROUND:**

Regulations in 2021 required the addition of a volatility reduction agent (VRA) to dicamba spray mixtures for postemergence weed control. Understanding the impact of VRAs on weed control, droplet dynamics, and spray pH is essential.

**RESULTS:**

Adding glyphosate to dicamba decreased the solution pH by 0.63 to 1.85 units. Across locations, potassium carbonate increased the tank‐mixture pH by 0.85 to 1.65 units while potassium acetate raised the pH by 0.46 to 0.53 units. Glyphosate and dicamba in tank‐mixture reduced Palmer amaranth control by 14 percentage points compared to dicamba alone and decreased barnyardgrass control by 12 percentage points compared to glyphosate alone 4 weeks after application (WAA). VRAs resulted in a 5‐percentage point reduction in barnyardgrass control 4 WAA. Common ragweed, common lambsquarters, and giant ragweed control were unaffected by herbicide solution 4 WAA. Dicamba alone produced a larger average droplet size and had the fewest driftable fines (% volume < 200 μm). Potassium acetate produced a larger droplet size than potassium carbonate for D_v0.1_ and D_v0.5_. The addition of glyphosate to dicamba decreased droplet size from the entire spray droplet spectrum (D_v0.1_, D_v0.5_, D_v0.9_).

**CONCLUSION:**

A reduction in spray pH, droplet size, and weed control was observed from mixing dicamba and glyphosate. It may be advisable to avoid tank‐mixtures of these herbicides and instead, apply them sequentially to maximize effectiveness. VRAs differed in their impacts on spray solution pH and droplet dynamics, but resulted in a minimal negative to no impact on weed control. © 2022 The Authors. *Pest Management Science* published by John Wiley & Sons Ltd on behalf of Society of Chemical Industry.

## Graphical abstract

Dicamba and glyphosate spray solution pH, droplet dynamics (size and velocity), and weed control as impacted by recent federally mandated volatility reduction agents (VRAs) were evaluated across a broad geography.

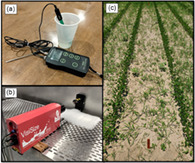

## 1 INTRODUCTION

The recent introduction of transgenic soybean [*Glycine max* (L.) Merr.] cultivars with stacked herbicide resistance traits to dicamba and glyphosate herbicides,^1^, ^2^ offered growers an additional tool for the management of herbicide‐resistant weeds. This commercialization was followed by an increase in both the areas planted with dicamba‐resistant crops and the amount of dicamba applied to control glyphosate‐resistant weeds. Dicamba off‐target movement became a major concern^3^, ^4^, ^5^ following the rise in within‐season applications of the herbicide^6^ with approximately 1.5 million hectares of soybean fields injured in 2017.^7^ Factors known to favor dicamba off‐target movement include dicamba formulations, spray solutions/tank‐mix partners, solution pH, droplet size, droplet velocity, and environmental conditions during and following applications.^8^, ^9^, ^10^, ^11^

Different dicamba formulations exist with the volatile dicamba acid^12^ formulated as a salt with either organic amine bases [dimethylamine (DMA) or diglycolamine (DGA)] or with a metal cation (such as sodium) that neutralizes the dicamba acid and decreases volatility.^8^ DMA dicamba salt is more volatile than the DGA dicamba salt.^13^ The commercialization of dicamba‐resistant crops was accompanied by the development of lower volatility formulations namely XtendiMax® with VaporGrip® technology (Bayer Crop Science, St. Louis, MO), Tavium® with VaporGrip® technology (Syngenta Crop Protection, Greensboro, NC), and Engenia® (BASF, Research Triangle Park, NC).^14^ XtendiMax® and Tavium® are formulated as the DGA salt with an acetic acid‐acetate buffering system (VaporGrip®) for a greater decrease of the volatility potential by scavenging protons in the spray solution.^15^ On the other hand, the Engenia® formulation contains a novel dicamba salt, N,N‐Bis‐(3‐aminopropyl) methylamine (BAPMA).^11^ Unfortunately, under certain conditions, these new formulations of dicamba have volatilized and moved to non‐target areas.^16^, ^17^, ^18^

The use of herbicides targeting multiple sites‐of‐action for managing herbicide‐resistant weeds is among the best recommended management practices.^19^ These herbicides are used either sequentially,^20^, ^21^ in pre‐mixtures,^22^, ^23^ or as tank‐mixtures.^21^, ^24^ Glyphosate is often used in combination with dicamba not only to manage glyphosate‐resistant weeds but also to broaden the weed control spectrum.^25^, ^26^ Approximately 80% of survey respondents in Nebraska used dicamba in a mixture with glyphosate for postemergence weed control in dicamba‐resistant soybean.^16^ However, agricultural glyphosate formulations had previously been reported to decrease the pH of a tank‐mixture with dicamba,^11^, ^27^ thereby raising the dicamba volatility potential.^8^ Label guidelines recommend avoidance of spray mixtures with pH values lower than 5.0 and the addition of buffering agents under such circumstances.^11^ In addition, federal regulations in 2021 required the addition of a volatility reduction agent (VRA) to dicamba spray mixtures to mitigate dicamba volatilization.

Spray droplet dynamics such as size and velocity are major parameters for chemical weed control. Droplet size is an essential factor that influences spray drift, deposition, spray coverage, canopy penetration, and biological efficacy^28^, ^29^, ^30^, ^31^ with droplets having diameters less than 200 μm showing the highest spray drift potential.^32^ Various application parameters are known to impact spray droplet size including spray mixtures.^33^, ^34^, ^35^ Creech *et al.*,^33^ reported an 11% decrease in spray solution droplet size when glyphosate was used in comparison to water. According to Sijs *et al.*,^36^ adding certain surfactant‐based adjuvants to an agricultural spray slightly reduced the volume median droplet size. Droplet velocity also plays a crucial role in spray particle drift.^37^, ^38^ An increase in vertical droplet velocity and a decrease in horizontal velocity were previously reported to reduce drift potential.^39^ Stopping distances are increased by greater exit velocities while smaller droplets, with lower terminal velocities, resulted in greater leaf adhesion.^31^, ^40^ Smaller droplets, with a lower terminal velocity, were reported to present a greater deposition on vertical plant surfaces and greater leaf retention.^41^ The reduction in drift potential due to an increase in spray droplet size has the consequence to decrease droplet velocity and consequently limit the potential of droplets to bounce or shatter.^10^

Only two VRAs, potassium acetate (VaporGrip® Xtra Agent, Bayer Crop Science, St. Louis, MO) and potassium carbonate (Sentris, BASF, Research Triangle Park, NC), were available for use with dicamba in 2021.^42^ It should be noted that although multiple commercial VRA products containing potassium acetate (VaporGrip® Xtra Agent) existed, as of 2021, all products were the same formulation. Although these spray additives were federally mandated, the impact of VRAs on spray solution pH, droplet dynamics (size and velocity), and weed control from dicamba and glyphosate spray solutions was unclear. Therefore, the objective of this study was to evaluate the impact of VRAs on spray solution pH, droplet dynamics, and weed control from dicamba (Engenia®) and glyphosate (Roundup PowerMax II) spray solutions across multiple soybean production areas in the United States.

## 2 MATERIALS AND METHODS

### 2.1 Spray solution pH

The pH of dicamba [Engenia® (BASF, Research Triangle Park, NC)] and glyphosate [Roundup PowerMax II (Bayer CropScience, St. Louis, MO)] alone and in mixture as affected by VRAs was evaluated in the laboratory at three locations (Table 1) [the Arlington Agricultural Research Station (AARS) near Arlington, WI, the Rohwer Research Station (RRS) near Rohwer, AR, and the Throckmorton Purdue Agricultural Center (TPAC) near Lafayette, IN]. The treatment design was a two‐factor factorial experiment using three levels of herbicides [dicamba (560 g ae ha^−1^), glyphosate (1261 g ae ha^−1^), dicamba (560 g ae ha^−1^) + glyphosate (1261 g ae ha^−1^)] and three levels of volatility reduction agents [None, potassium carbonate (0.6 L ha^−1^) (Sentris), potassium acetate (1.5 L ha^−1^) (VaporGrip® Xtra Agent)]. A nontreated control (water alone) was added as a baseline pH reference. Prior to measurements, pH meters were calibrated using the National Institute of Standards and Technology (Gaithersburg, MD) buffer standards.^11^ Spray solutions were prepared by mixing spray water from each location with herbicide and VRA treatments to a total volume of 100 mL with a carrier volume rate of 140 L ha^−1^.^11^ The pH of each solution was measured after a thorough agitation of the solution. An Oakton pHTestr® 50 Waterproof Pocket pH Tester Premium 50 Series probe (Oakton Instruments, Vernon Hills, IL) was used at the AARS, a Milwaukee MW102 PRO pH/Temperature Meter (Milwaukee Instruments, Rocky Mount, NC) was used at the RRS, and a Mettler Toledo FE20 pH meter (Mettler‐Toledo, LLC, Columbus, OH) was used at the TPAC. Three readings were taken for each treatment at each location with the electrode rinsed between measurements.^11^

**Table 1:** Descriptions of experimental conditions for the field experiments conducted at three locations in 2021 [the Arlington Agricultural Research Station (AARS) near Arlington, WI, the Rohwer Research Station (RRS) near Rohwer, AR, and the Throckmorton Purdue Agricultural Center (TPAC) near Lafayette, IN] to evaluate the impact of VRAs on weed control from dicamba (Engenia®) and glyphosate (Roundup PowerMax II)

Experimental conditions	AARS*	RRS*	TPAC*
Soil characteristics	Plano silt loam 3.8% OM, pH 6.6, 10 CEC	Silt loam pH 7.0, 11 CEC	Toronto‐Millbrook silt‐loam 3.1% OM, pH 6.2, 115 CEC
Soybean variety	AG20XF1	AG46XF0	AG29XF1
Planting details	June 1, 2021 311 111 seeds ha^−1^ 76 cm row spacing	May 24, 2021 327 645 seeds ha^−1^ 97 cm row spacing	May 26, 2021 311 111 seeds ha^−1^ 76 cm row spacing
Herbicide application	June 28, 2021 V3 growth stage	June 17, 2021 V3 growth stage	June 11, 2021 VC growth stage
Primary weeds*	Common ragweed Common lambsquarters Giant foxtail	Palmer amaranth Barnyardgrass	Giant ragweed
Weather conditions^†^	Temp: 23.3 °C RH: 80% Wind speed: 0.45–1.3 m s^−1^	Temp: 31.7 °C RH: 45% Wind speed: 2.1 m s^−1^.	Temp: 32.5 °C RH: 43% Wind speed: 1.8 m s^−1^
GPS coordinates	43°18'9.47″ N 89°20'43.32″ W	33°51'49.115″ N 92°19'45.609″ W	40°17'48.264″ N 86°54'13.039″ W

* Scientific names: Common ragweed, *Ambrosia artemisiifolia* L.; Common lambsquarters, *Chenopodium album* L.; Giant foxtail, *Setaria faberi* Herrm.; Palmer amaranth, *Amaranthus palmeri* S. Wats.; Barnyardgrass, *Echinochloa crus‐galli* (L.) P. Beauv.; Giant ragweed, *Ambrosia trifida* L.

^†^ Temp: average air temperature, RH: relative humidity and average wind speed at the time of application.

### 2.2 Field experiments

Field experiments were conducted in 2021 at the same three locations in which the spray solution pH was measured (Table 1) to evaluate the impact of VRAs on weed control from dicamba (Engenia®) and glyphosate (Roundup PowerMax II). Conventional tillage was used for field preparation at all locations. At the AARS, glyphosate (Roundup PowerMax II) was applied 2 days after planting at 1260 g ae ha^−1^ + 1% dry ammonium sulfate by weight to control weeds that had emerged after the spring tillage. Randomized complete block designs were established using four replications and an XtendFlex™ soybean adapted to each location was planted. The treatment design was a two‐factor factorial experiment with the same treatments as the spray pH measurements. A nontreated control was added as a reference for weed control evaluations. Herbicides were applied with CO_2_ backpack sprayers calibrated to deliver 140 L ha^−1^ and equipped with TTI 110015 nozzles. *S*‐metolachlor [Dual Magnum (Syngenta Crop Protection, Greensboro, NC)] was applied at 1420 g ai ha^−1^ for residual control of secondary weed flushes following treatments at all locations.

Visual assessments of weed control were taken on a scale of 0 to 100% (0 being no control and 100 being complete control) 2 and 4 weeks after herbicide application (WAA). Additionally, density counts per species were recorded 4 WAA at the AARS and RRS. Weed species that survived herbicide treatments were counted and recorded from two 0.25 m^2^ quadrats randomly selected per plot. Next, weeds counted in each quadrat were clipped at the soil surface, placed in separate paper bags per weed species, dried to constant biomass, and weighed. Weed biomass data were converted into percent biomass reduction compared to the nontreated control for each replication using Eqn (1)^43^, ^44^

(1) $$\text{Biomass reduction}\;\left(\%\right)=\left[\frac{\left(C-B\right)}{C}\right]*100$$

where C is the biomass of the nontreated control and B is the biomass of the treated plot.

### 2.3 Droplet size and velocity experiment

**Figure 1:**
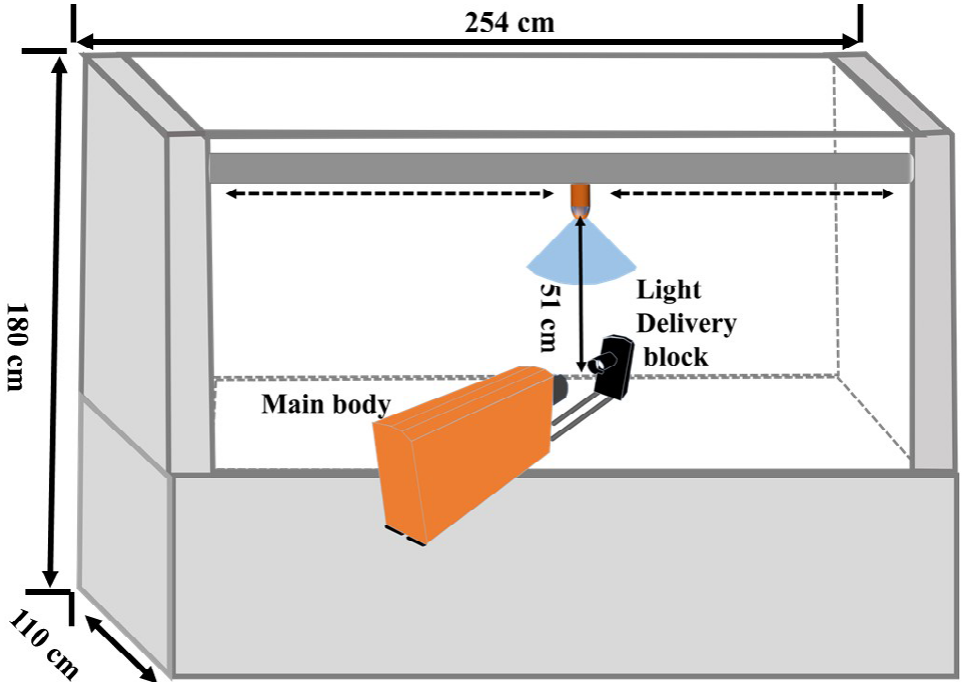
Illustration of equipment setup for droplet size and velocity measurements using the VisiSize P15 Portable Particle/Droplet Image Analysis System installed in the Generation 4 Research Track Sprayer equipped with a single TTI 110015 nozzle.

A laboratory experiment was conducted at the Lonoke Extension Center near Lonoke, AR to evaluate the impact of VRAs on the droplet size and velocity of dicamba and glyphosate spray solutions. The experiment was a completely randomized design with three replications and measurements were recorded similarly to previous droplet velocity research.^10^ Measurements were made using the VisiSize P15 Portable Particle/Droplet Image Analysis System (Oxford Lasers, Imaging Division, Oxford, UK). Prior to measurement, the VisiSize P15 components were aligned and calibrated according to manufacturer's recommendations. The system was installed within a Generation 4 Research Track Sprayer (Devries Manufacturing, Hollandale, MN) equipped with a single TTI 110015 nozzle (Fig. 1). The long axis of the nozzle was oriented perpendicularly to the axis of the diffused light aperture and the camera window/spray guard. The distance between the nozzle tip and the measurement zone was set to 51 cm to allow droplet size and velocity measurements to coincide with the spray boom height used in the field experiments. The VisiSize P15 system measured spray velocity by taking sequential paired images 10 μs apart. Herbicide treatments were mixed for a carrier volume rate of 140 L ha^−1^. The spray chamber was operated at 276 kPa and traversed at 0.22 m s^−1^ to allow for sampling of spray droplets from the entire spray plume. Data acquisition was set to measure diameter and velocity of 2500 individual droplets per replication, giving a total of 7500 droplets measured per treatment.

### 2.4 Data analyses

Broadleaf and grass control, weed density, and biomass reduction were compared among herbicides and VRAs. Sites were considered fixed effects while blocks nested within sites were considered random effects. Weed control and biomass reduction data were subjected to ANOVA using the GLIMMIX procedure in SAS version 9.4 (SAS Institute Inc, Cary, NC, United States) assuming a beta distribution.^45^ Weed density data were subjected to ANOVA using the GLIMMIX procedure in SAS assuming a Poisson distribution.^45^ Treatment means were separated using Fisher's protected LSD (α = 0.1). The use of the Colby's equation allowed the type of interaction between glyphosate and dicamba in tank‐mixture (additive, synergistic, or antagonistic) to be determined by calculating expected control [Eqn (2)]^46^ and using a t‐test to compare expected and observed control^43^ within SAS.

(2) $$E=\left(X+Y\right)-\frac{\mathrm{XY}}{100}$$

where E is the expected weed control when glyphosate and dicamba were applied in tank‐mixture, X and Y are the observed weed control when glyphosate and dicamba were applied alone, respectively. According to Colby^46^ a synergistic combination is determined by an observed response greater than expected, an antagonistic combination determined by an observed response smaller than expected, and an additive combination determined by equal observed and expected responses. The Colby's equation was used to estimate expected control solely when both herbicides can have activity on a given weed (*e.g*., no grass control from dicamba).^47^, ^48^

Droplet size data (D_v0.1_, D_v0.5_, and D_v0.9_, representing 10, 50, and 90% of the spray volume comprised of droplets of a smaller diameter, respectively), relative span [(RS), Eqn (3)], and droplet velocity data were subjected to ANOVA using the GLIMMIX procedure in SAS assuming a gamma distribution.^49^

(3) $$\mathrm{RS}=\frac{{\mathrm{Dv}}_{0.9}-{\mathrm{Dv}}_{0.1}}{{\mathrm{Dv}}_{0.5}}$$

The percent of the spray volume containing droplets 200 μm in diameter and smaller, referred to as driftable fines, were predicted using the Rosin–Rammler (RR) equation [Eqn (4)].^50^

(4) $$V\left(d\right)=100-100*\exp\left(-{\left(\frac{d}{c}\right)}^{m}\right)$$

where *V* is the cumulative % volume of droplets with the diameter lower than a certain value (*d*). *c* is the characteristic droplet diameter, defined as the diameter at which the cumulative volume fraction is 63.2%. *m* is a constant indicating the uniformity of the distribution.^50^

The three parameter log‐logistic model [Eqn (5)] was fit to droplet size and velocity paired measurements data using non‐linear least squares regression (nls) in R version 4.0.0.^51^

(5) $$Y=\frac{d}{1+\exp\left[b\left(\text{logx}-\text{loge}\right)\right]}$$

where Y is the droplet exit velocity (m s^−1^), b is the relative slope around e, d is the upper limit (m s^−1^), e is the inflection point, and x is the droplet size (μm).^10^

The goodness of fit of each model was assessed using root mean square error [RMSE, Eqn (6)] and modeling efficiency [ME, Eqn (7)].^52^

(6) $$\text{RMSE}=\sqrt{\frac{1}{N}\sum_{i=1}^{N}{\left(Y_{i}-{\hat{Y}}_{i}\right)}^{2}}$$

where Y_*i*_ is the measured value and ${\hat{Y}}_{i}$ is the corresponding value predicted by the model. N is the total number of observations. Smaller RMSE values indicate a better model fit to data with a perfect fit indicated by RMSE values of 0.^52^, ^53^

(7) $$\mathrm{ME}=1-\frac{\sum_{i=1}^{N}{\left(Y_{i}-{\hat{Y}}_{i}\right)}^{2}}{\sum_{i=1}^{N}{\left(Y_{i}-\bar{Y}\right)}^{2}}$$

where $\bar{Y}$ is the mean observed value. Closer values to 1 are an indication of more accurate predictions.^53^

## 3 RESULTS

### 3.1 Spray solution pH

The pH of the spray water used was alkaline at AARS and TPAC with values of 7.56 and 7.41, respectively, while at the RRS the water was acidic (pH 5.95) (Table 2). Spray solutions with dicamba alone had pH values between 5.32 and 6.69 while spray solutions with glyphosate alone had pH values between 4.67 and 4.95. The addition of potassium carbonate to dicamba induced a 1.20 to 3.26 pH unit increase, whereas the addition of potassium acetate to dicamba induced a 0.16 to 0.31 pH unit increase. The addition of either VRA increased glyphosate spray solution pH (Table 2). Potassium carbonate induced between 1.18 and 1.26 pH unit increase when added to glyphosate while potassium acetate provoked between 0.40 and 0.54 pH unit increase when added to glyphosate. Adding glyphosate to dicamba decreased the solution pH by 1.85, 0.63, and 1.77 pH units at the AARS, RRS, and TPAC, respectively, leading to spray solution pH values below 5.0 at all locations. However, both VRAs consistently increased the pH of dicamba and glyphosate in tank‐mixture. Potassium carbonate increased the pH of the tank‐mixture by 0.85, 1.25, and 1.36 pH units while potassium acetate stabilized pH values close to the starting pH with 0.46, 0.53, and 0.52 pH units increase at the AARS, RRS, and TPAC, respectively.

**Table 2:** Spray solution pH of dicamba and glyphosate alone and in mixture as affected by volatility reduction agents (VRA) across three locations in the United States

Herbicide	VRA	AARS*	RRS*	TPAC*
		^___________________^pH (sd^†^)^__________________^		
None	None	7.56 (0.07)	5.95 (0.01)	7.41 (0.03)
Glyphosate	None	4.92 (0.01)	4.67 (0.02)	4.95 (0.01)
	Potassium carbonate	6.10 (0.01)	5.88 (0.02)	6.21 (0.01)
	Potassium acetate	5.32 (0.01)	5.21 (0.02)	5.48 (0.02)
Dicamba	None	6.66 (0.01)	5.32 (0.01)	6.69 (0.01)
	Potassium carbonate	9.55 (0.03)	6.52 (0.01)	9.95 (0.01)
	Potassium acetate	6.83 (0.04)	5.63 (0.02)	6.85 (0.01)
Dicamba + Glyphosate	None	4.81 (0.01)	4.69 (0.00)	4.92 (0.01)
	Potassium carbonate	5.66 (0.02)	5.94 (0.00)	6.28 (0.01)
	Potassium acetate	5.27 (0.01)	5.22 (0.01)	5.44 (0.01)

* AARS: the Arlington Agricultural Research Station near Arlington, WI; RRS: Rohwer Research Station near Rohwer, AR; TPAC: the Throckmorton Purdue Agricultural Center near Lafayette, IN.

^†^ sd is the standard deviation.

### 3.2 Weed control, weed density and aboveground biomass reduction

For broadleaf and grass control 2 and 4 WAA and for broadleaf and grass density and biomass reduction 4 WAA, the site‐by‐herbicide‐by‐VRA and herbicide‐by‐VRA interactions were not significant (Table 3). The site‐by‐herbicide interaction was significant for broadleaf and grass control 2 and 4 WAA, and broadleaf and grass density and biomass reduction 4 WAA. Also, the site‐by‐VRA interaction was significant for grass control 4 WAA. Therefore, results are presented for each individual site and by individual weed species within site.

**Table 3:** Analysis of variance output (Pr > F) for visual estimations of weed control, biomass reduction, and density of pooled broadleaf and grass weed species across three locations in the United States

	2 WAA Control		4 WAA Control		Biomass reduction		Density	
	Broadleaf	Grass	Broadleaf	Grass	Broadleaf	Grass	Broadleaf	Grass
Site	<0.0001	0.0002	<0.0001	<0.0001	<0.0001	0.9611	<0.0001	<0.0001
Herbicide	<0.0001	<0.0001	<0.0001	<0.0001	<0.0001	<0.0001	<0.0001	<0.0001
Site*Herbicide	**<0.0001**	**<0.0001**	**<0.0001**	**<0.0001**	**<0.0001**	**0.0096**	**<0.0001**	**<0.0001**
VRA	0.7073	0.2679	0.5991	0.0661	0.8983	0.5246	0.5564	0.3334
Site*VRA	0.9435	0.2679	0.6857	**0.0737**	0.9702	0.4819	0.5442	0.4187
Herbicide*VRA	0.9953	0.2637	0.9327	0.4993	0.9788	0.4748	0.5050	0.3762
Site*Herbicide*VRA	0.9999	0.2637	0.9498	0.5255	0.9977	0.5005	0.3875	0.5210

Across VRA treatments, dicamba provided the least control of both common ragweed and common lambsquarters 2 WAA at the AARS (Table 4). However, all herbicides provided identical control of common ragweed and common lambsquarters 4 WAA. Analysis of Colby's equation showed additive interactions of glyphosate and dicamba for common ragweed and common lambsquarters control. VRA did not influence giant foxtail control (Table 4). All treatments provided identical biomass reduction (Table 5) and weed density (Table 6) of common ragweed and common lambsquarters. Glyphosate alone and in tank‐mixture with dicamba provided a similar density of giant foxtail (Table 6). VRAs did not impact weed control, biomass reduction, or weed density at the AARS (Tables 4, 5, and 6).

**Table 4:** Control (%) of common ragweed (*Ambrosia artemisiifolia* L.) (CRA), common lambsquarters (*Chenopodium album* L.) (CLA), and giant foxtail (*Setaria faberi* Herrm.) (GFO) using dicamba and glyphosate alone and in tank‐mixture as affected by volatility reduction agent (VRAs) at the Arlington Agricultural Research Station near Arlington, WI. The main effects of herbicide averaged across VRAs and VRA averaged across herbicides are presented as no significant interaction was observed

	CRA*				CLA				GFO	
	2 WAA		4 WAA		2 WAA		4 WAA		2 WAA	4 WAA
	O^†^	E	O	E	O	E	O	E		
Herbicide										
Dicamba	95b	‐	100a	‐	82b	‐	98a	‐	0b	0b
Glyphosate	100a	‐	99a	‐	100a	‐	100a	‐	100a	100a
Dicamba + glyphosate^‡^	100aA	100A	100aA	100A	100aA	100A	100aA	100A	100a	100a
VRA										
None	98		100		94		99		67	67
Potassium carbonate	98		100		94		99		67	67
Potassium acetate	99		100		95		100		67	67
	*Pr > F*		*Pr > F*		*Pr > F*		*Pr > F*		***Pr > F***	***Pr > F***
Herbicide	<0.0001		0.139		<0.0001		0.1997		<0.0001	<0.0001
VRA	0.5588		0.118		0.7992		0.5383		0.6127	0.3827
Herbicide*VRA	0.3550		0.1569		0.9211		0.6421		0.3168	0.4269

* Abbreviations: WAA, weeks after application; O, observed weed control rate; E, expected weed control rate for glyphosate and dicamba in tank‐mixture calculated using the Colby's equation.

^†^ Means within a column followed by different lowercase letters are different based on Fisher's protected LSD (α = 0.1).

^‡^ Observed and expected control rate within a row (for the same species and evaluation date) followed by the same uppercase letter are not different based on the t‐test (α = 0.1).

**Table 5:** Biomass reduction (%) of common ragweed (*Ambrosia artemisiifolia* L.) (CRA), common lambsquarters (*Chenopodium album* L.) (CLA), giant foxtail (*Setaria faberi* Herrm.) (GFO), Palmer amaranth (*Amaranthus palmeri* S. Wats.) (PAM), barnyardgrass [*Echinochloa crus‐galli* (L.) P. Beauv.] (BYG), and giant ragweed (*Ambrosia trifida* L.) (GRA) using dicamba and glyphosate alone and in tank‐mixture as affected by volatility reduction agent (VRA). The main effects of herbicide averaged across VRAs and VRA averaged across herbicides are presented as no significant interaction was observed

	AARS*			RRS*	
	CRA^†^	CLA	GFO	PAM	BYG
Herbicide					
Dicamba	100	100	0b	100a	0b
Glyphosate	100	100	100a	0c	100a
Dicamba + glyphosate	100	100	99a	51b	92a
VRA					
None	100	100	67	53	69
Potassium carbonate	100	100	66	50	67
Potassium acetate	100	100	67	47	63
	*Pr > F*	*Pr > F*	*Pr > F*	*Pr > F*	*Pr > F*
Herbicide	0.1312	0.999	<0.0001	<0.0001	0.0017
VRA	0.1458	0.999	0.3827	0.8267	0.5636
Herbicide*VRA	0.1138	0.999	0.4269	0.9403	0.2321

* AARS: the Arlington Agricultural Research Station near Arlington, WI; RRS: Rohwer Research Station near Rohwer, AR.

^†^ Means within a column followed by different lowercase letters are different based on Fisher's protected LSD (α = 0.1).

**Table 6:** Density (plants m^−2^) of common ragweed (*Ambrosia artemisiifolia* L.) (CRA), common lambsquarters (*Chenopodium album* L.) (CLA), giant foxtail (*Setaria faberi* Herrm.) (GFO), Palmer amaranth (*Amaranthus palmeri* S. Wats.) (PAM), barnyardgrass [*Echinochloa crus‐galli* (L.) P. Beauv.] (BYG), giant ragweed (*Ambrosia trifida* L.) (GRA) using dicamba and glyphosate alone and in tank‐mixture as affected by volatility reduction agent (VRA). The main effects of herbicide averaged across VRAs and VRA averaged across herbicides are presented as no significant interaction was observed

	AARS*			RRS	
	CRA^†^	CLA	GFO	PAM	BYG
Herbicide					
Dicamba	0	0	4a	0c	108a
Glyphosate	0	0	0b	52a	0c
Dicamba + glyphosate	0	0	0b	11b	9b
VRA					
None	0	0	1	24	34
Potassium carbonate	0	0	1	24	37
Potassium acetate	0	0	2	16	44
	*Pr > F*	*Pr > F*	*Pr > F*	*Pr > F*	*Pr > F*
Herbicide	0.9999	0.9999	<0.0001	<0.0001	<0.0001
VRA	0.9999	0.9999	0.5073	0.2839	0.3818
Herbicide*VRA	0.9999	0.9999	0.5317	0.1056	0.4550

* AARS: the Arlington Agricultural Research Station near Arlington, WI; RRS: Rohwer Research Station near Rohwer, AR.

^†^ Means within a column followed by different lowercase letters are different based on Fisher's protected LSD (α = 0.1).

Glyphosate alone provided the least control of Palmer amaranth 2 and 4 WAA (Table 7) at the RRS while dicamba alone provided the highest control of Palmer amaranth 4 WAA. Analysis of Colby's equation revealed an additive interaction for Palmer amaranth control 2 WAA when dicamba and glyphosate were applied in a tank‐mixture. However, an antagonistic interaction was detected 4 WAA. Across VRAs, glyphosate and dicamba in tank‐mixture reduced Palmer amaranth control by 14 percentage points 4 WAA compared to dicamba alone. Glyphosate alone provided the greatest control of barnyardgrass 2 and 4 WAA. Glyphosate and dicamba in tank‐mixture decreased barnyardgrass control by 6 and 12 percentage points 2 and 4 WAA, respectively, compared to glyphosate alone. Across herbicides, glyphosate alone provided the greatest aboveground biomass reduction of barnyardgrass (Table 5). The addition of glyphosate to the mixture decreased the effectiveness of dicamba as Palmer amaranth biomass reduction was reduced by 49‐percentage points compared to dicamba alone which is consistent with the antagonistic interaction observed in weed control (Tables 5 and 7). Similarly, Palmer amaranth density increased from 0 plants m^−2^ (dicamba alone) to 11 plants m^−2^ with the tank‐mixture of dicamba and glyphosate while barnyardgrass density increased form 0 plants m^−2^ (glyphosate alone) to 9 plants m^−2^ (Table 6). VRAs induced a three‐percentage point reduction in barnyardgrass control 4 WAA at the RRS (Table 7) but did not influence biomass reduction nor weed density (Tables 5 and 6).

**Table 7:** Control (%) of Palmer amaranth (*Amaranthus palmeri* S. Wats.) (PAM) and barnyardgrass [*Echinochloa crus‐galli* (L.) P. Beauv.] (BYG) using dicamba and glyphosate alone and in tank‐mixture as affected by volatility reduction agent (VRAs) at the Rohwer Research Station (RRS) near Rohwer, AR. The main effects of herbicide averaged across VRAs and VRA averaged across herbicides are presented as no significant interaction was observed

	PAM*				BYG*	
	2 WAA		4 WAA		2 WAA	4 WAA
	O^†^	E	O	E	O	O
Herbicide						
Dicamba	95a	‐	99a	‐	0c	0c
Glyphosate	40b	‐	3c	‐	100a	95a
Dicamba + glyphosate^‡^	93aA	97A	85bB	99A	94b	83b
VRA						
None	75	‐	62	‐	66	61a
Potassium carbonate	75	‐	61	‐	64	58b
Potassium acetate	78	‐	65	‐	65	58b
	*Pr > F*		*Pr > F*		*Pr > F*	*Pr > F*
Herbicide	<0.0001		<0.0001		<0.0001	0.0002
VRA	0.4561		0.4223		0.2749	0.0788
Herbicide*VRA	0.8806		0.5527		0.2760	0.2017

* Abbreviations: WAA, weeks after application; O, observed weed control rate; E, expected weed control rate for glyphosate and dicamba in tank‐mixture calculated using the Colby's equation.

^†^ Means within a column followed by different lowercase letters are different based on Fisher's protected LSD (α = 0.1).

^‡^ Observed and expected control rate within a row (for the same species and evaluation date) followed by the same uppercase letter are not different based on the t‐test (α = 0.1).

Glyphosate alone provided the least control of giant ragweed 2 WAA (Table 8) at the TPAC. Analysis of Colby's equation showed an additive interaction of dicamba and glyphosate in tank‐mixture. However, giant ragweed control was not impacted by herbicides 4 WAA as all herbicide solutions provided 100% control equivalent to 100% biomass reduction and a density of 0 plants m^−2^. Additionally, VRAs did not impact weed control, biomass reduction, or weed density at the TPAC (Table 8).

**Table 8:** Control (%) of giant ragweed (*Ambrosia trifida* L.) (GRA) using dicamba and glyphosate alone and in tank‐mixture as affected by volatility reduction agent (VRAs) at the Throckmorton Purdue Agricultural Center near Lafayette, IN

	GRA			
	2 WAA*		4 WAA	
	O^†^	E	O	E
Herbicide				
Dicamba	97a	‐	100	‐
Glyphosate	93b	‐	100	‐
Dicamba + glyphosate^‡^	97aA	100A	100A	100A
VRA				
None	96		100	‐
Potassium carbonate	96		100	‐
Potassium acetate	96		100	‐
	*Pr > F*		*Pr > F*	
Herbicide	<0.0001		0.999	
VRA	0.8998		0.999	
Herbicide*VRA	0.9429		0.999	

* Abbreviations: WAA, weeks after application; O, observed weed control rate; E, expected weed control rate for glyphosate and dicamba in tank‐mixture calculated using the Colby's equation.

^†^ Means within a column followed by different lowercase letters are different based on Fisher's protected LSD (α = 0.1).

^‡^ Observed and expected control rate within a row (for the same species and evaluation date) followed by the same uppercase letter are not different based on the t‐test (α = 0.1).

### 3.3 Droplet size and velocity

**Figure 2:**
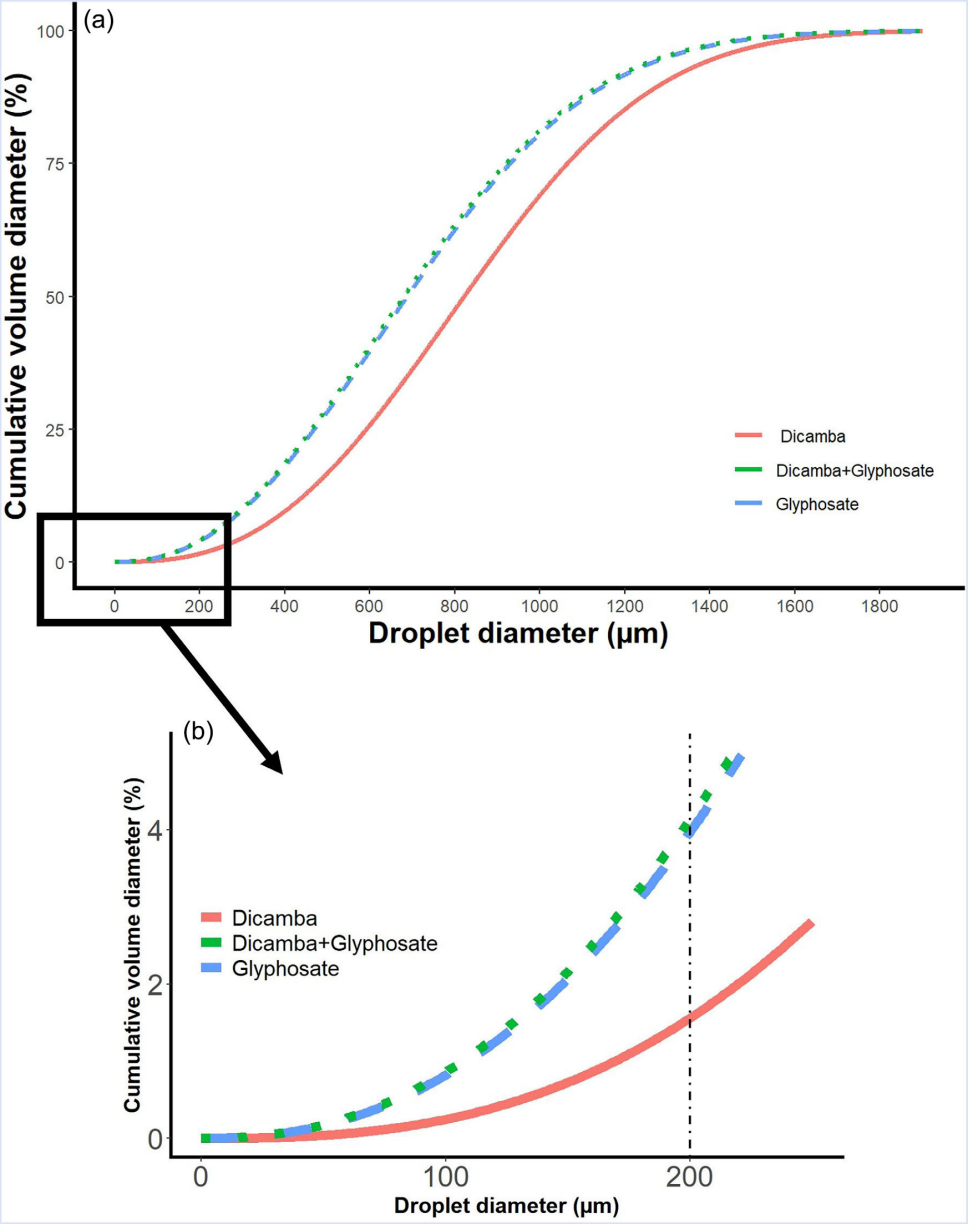
Cumulative volumetric droplet size distribution of dicamba, glyphosate, and dicamba + glyphosate spray solutions, averaged across volatility reduction agents.

The herbicide‐by‐VRA interaction for the D_v0.1_, D_v0.5_, D_v0.9_, and the relative span were not significant (Table 9). Across VRAs, dicamba alone produced a larger droplet size from the entire spray droplet spectrum at 51 cm from the nozzle tip. At the same time, it produced the smallest relative span. Across herbicides, potassium acetate produced a larger droplet size than potassium carbonate for D_v0.1_ and D_v0.5_ (Table 9). The RR equation provided a good fit to droplet size data of all spray solutions with RMSE values between 1.40 and 4.58 and modeling efficiency (ME) values were 0.98–0.99. Dicamba alone had the smallest percentage of droplets less than 200 μm in diameter, referred to as ‘driftable’ fines (Table 9). The addition of glyphosate to dicamba decreased droplet size from the entire spray droplet spectrum (D_v0.1_, D_v0.5_, D_v0.9_) and increased the relative span. Moreover, it shifted the RR curve to the left and increased the drift potential of the spray solution by increasing the percentage of driftable fines by 2.7 times regardless of the addition of VRAs (Fig. 2).

**Table 9:** Droplet size distribution parameters for spray solutions of dicamba and glyphosate alone and in tank‐mixture as affected by volatility reduction agent (VRA). Driftable fines (predicted % of droplets smaller than 200 μm), root mean square error (RMSE) and modeling efficiency (ME) of the Rosin‐Rammler model fitted to droplet size distribution data

	D_v0.1_ *	D_v0.5_ *	D_v0.9_ *	RS^†^	Driftable fines*	RMSE	ME
	^____________^μm^‡^ ^____________^				%		
Herbicide							
Water alone	391	807	1204	1.01	1.52	1.64	0.99
Dicamba	391a	846a	1298a	1.07b	1.56	1.40	0.99
Glyphosate	285b	713b	1140b	1.20a	3.97	2.33	0.99
Dicamba+Glyphosate	286b	702b	1127b	1.20a	4.13	2.28	0.99
VRA							
None	320ab	758ab	1176	1.13	3.07	3.48	0.99
Potassium carbonate	315b	741b	1184	1.18	3.47	4.58	0.98
Potassium acetate	328a	763a	1205	1.16	3.05	3.48	0.99
		*Pr > F*					
Herbicide	<0.0001	<0.0001	<0.0001	0.0010			
VRA	0.0657	0.0775	0.4055	0.4246			
Herbicide*VRA	0.1038	0.3419	0.1036	0.6297			

* D_V0.1_, D_V0.5_, D_V0.9_ are volume diameter (μm) below which smaller droplets represent 10%, 50% and 90% of the total volume, respectively. Driftable fines are defined as % spray volume containing droplets <200 μm in diameter.

^†^ RS is the relative span (dimensionless parameter used to measure the spread of the drop size in the spray and indicating the uniformity of the drop size distribution).

^‡^ Means within a column followed by different letters are different based on Fisher's protected LSD (α = 0.1).

**Figure 3:**
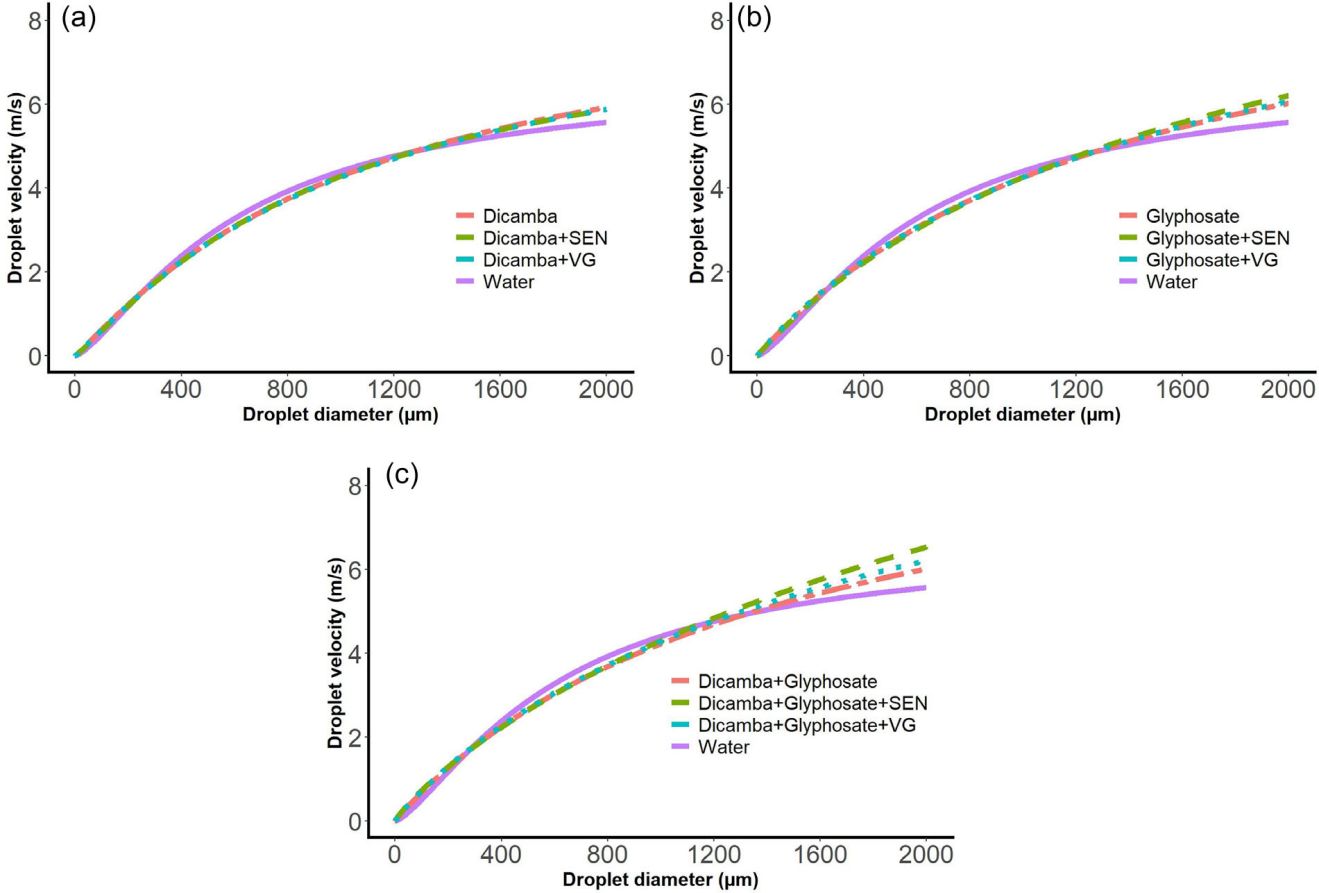
Droplet velocity as a function of droplet size for (A) dicamba, (B) glyphosate, and (C) dicamba + glyphosate spray solutions as affected by volatility reduction agents. Abbreviations: SEN, Sentris, potassium carbonate; VG, VaporGrip Xtra, potassium acetate.

The herbicide‐by‐VRA interaction for average and maximum velocity was significant (Table 10). The average velocity of droplets produced by potassium acetate was faster compared to that produced by both potassium carbonate and water when glyphosate was used. On the other hand, the maximum velocity of droplets produced by water was faster compared to that produced by both VRAs when glyphosate was used (Table 10). The 3‐parameter log logistic model provided a good fit to data with RMSE values ranging between 0.30 and 0.37. The modeling efficiency had values between 0.86 and 0.92. In all cases, an increase in droplet size induced an increase in droplet velocity until the plateau was reached (Fig. 3). According to the 3‐parameter log logistic model, the predicted velocity of 200 μm diameter spray droplets from highest to lowest followed the pattern dicamba + glyphosate > glyphosate > dicamba > water.

**Table 10:** Average, maximum and predicted droplet velocity for the spray solutions of dicamba and glyphosate alone and in tank‐mixture as affected by volatility reduction agents (VRA). Root mean square error (RMSE) and modeling efficiency (ME) for the 3‐parameter log‐logistic model fitted to droplet size and velocity data

Herbicide	VRA	Average velocity*	Maximum velocity*	Velocity of droplets			RMSE	ME
				200 μm	400 μm	500 μm		
		^___________________________________________^m s^−1____________________________________^						
Water	None	1.73	6.49	1.18	2.38	2.86	0.37	0.90
Dicamba	None	1.59a	5.35a	1.22	2.26	2.69	0.32	0.91
	Potassium carbonate	1.60a	5.19a	1.20	2.25	2.69	0.32	0.92
	Potassium acetate	1.59a	5.40a	1.21	2.25	2.68	0.32	0.92
Glyphosate	None	1.47b	5.39b	1.26	2.25	2.67	0.31	0.88
	Potassium carbonate	1.42b	4.78c	1.24	2.22	2.64	0.30	0.88
	Potassium acetate	1.53c	4.99c	1.29	2.27	2.69	0.32	0.88
Dicamba+Glyphosate	None	1.48d	5.07d	1.26	2.24	2.66	0.32	0.87
	Potassium carbonate	1.49d	4.97d	1.29	2.23	2.64	0.32	0.86
	Potassium acetate	1.51d	4.96d	1.30	2.27	2.68	0.32	0.87
		***Pr > F***	***Pr > F***					
Herbicide		<0.0001	0.0018					
VRA		0.0521	0.0065					
Herbicide*VRA		0.0505	0.0645					

* Means within a column followed by different letters are different based on Fisher's protected LSD (α = 0.1).

## 4 DISCUSSION

The range of pH values reported by this study is in alignment with values reported by previous research. In Wisconsin, the pH of water sources used by Striegel *et al.* ^11^ were between 7.45 and 7.70. In Arkansas, Butts *et al.* ^54^ reported spray water with pH values between 5.91 and 8.82 with 72% of water samples having pH > 7. Additionally, the pH of 82% of US groundwater sources reported by DeSimone *et al.* ^55^ varied between 6.5 and 8.0. The acidic water source at the RRS reduced the pH of all spray solutions compared to the other locations. The low pH obtained in dicamba‐glyphosate tank‐mixtures, and the pH increase after VRA addition to the spray mixture is consistent with previous research. Solution pH was increased following the addition of a pH buffer (MON 51817).^11^ According to Mueller and Steckel^27^ the addition of glyphosate to any dicamba formulation decreased spray solution pH; the final pH being a function of the water source's initial pH. Striegel *et al.* ^11^ also reported a decrease of spray solution to a pH below 5.0 when glyphosate was added to an auxin herbicide. By decreasing the pH of a tank‐mixture with dicamba (pH ∼ 4.5), agricultural glyphosate formulations increase the availability of protons at a lower pH. At this pH < 5.0, dicamba more promptly converts to the acid form that has very high vapor potential; thereby increasing its volatility potential.^8^ Dicamba is a weak acid (pKa of 1.87) with most products formulated as dicamba salts of organic amines.^12^ Once dissolved in water these dicamba salts of organic amines dissociate into dicamba anion and counterion with the dicamba anion able to combine with any available proton in solution to form the volatile dicamba acid according to the Henderson‐Hasselbalch equation relating a weak acid degree of dissociation to the pKa of the acid and the pH of the solution.^8^ The VRAs increase the pH of spray solutions by reducing protons number and availability with some containing acetate buffer and others being pH buffers.^8^ The pH buffers raise the spray tank pH and limit the number of protons while the acetate buffer reduces volatility by scavenging protons in the system.^8^ According to Abraham,^15^ the acetic acid‐acetate buffering system (pKa 4.75) scavenges extraneous protons that could be brought into the system from tank mixtures. The simultaneous dissociation of acetic acid‐acetate and dicamba salt in solution allows the buffering action of acetate to scavenge any extraneous protons from tank additives in the spray solution, thus maintaining the pH at or very close to the starting pH of the formulation, and therefore preventing the formation of dicamba acid.

Barnyardgrass density was less for glyphosate alone compared to glyphosate and dicamba in tank‐mixture. Similarly, Palmer amaranth density was less for dicamba alone compared to dicamba and glyphosate in tank‐mixture. These two weeds are among the most problematic weeds for modern agriculture. Palmer amaranth is a prolific seed producer with a single female plant having the ability to produce up to 600 000 seeds that can replenish the soil seedbank in a single generation.^56^ Likewise, barnyardgrass is also a prolific seed producer capable of producing up to 39 000 seeds plant^−1^ ^57^ that will increase the soil seedbank and compromise subsequent growing seasons.^58^ Both species have evolved resistance to a combined 9 herbicide sites‐of‐action.^59^ An important recommendation for management of herbicide‐resistant weeds is to avoid weed escapes to produce seeds.^19^ With an increase of Palmer amaranth density from 0 (dicamba alone) to 11 plants m^−2^ and barnyardgrass density from 0 (glyphosate alone) to 9 plants m^−2^, the tank‐mixture of dicamba and glyphosate allowed these two species to increase the soil seedbank; thereby compromising future weed control efforts. The antagonistic and additive interactions of dicamba and glyphosate observed in this study are consistent with previous research.^60^, ^61^, ^62^

Common ragweed, common lambsquarters and giant foxtail populations at the AARS, and giant ragweed populations at the TPAC were susceptible to both herbicides used in this research as revealed by the high level of control achieved by the herbicides 2 and 4 WAA. In contrast, Palmer amaranth populations at the RRS had a high level of resistance to glyphosate as revealed by the poor control observed and barnyardgrass has exhibited increased tolerance to glyphosate across Arkansas in recent years (Butts and Norsworthy, personal communication). Antagonistic interactions are a function of different factors including herbicide rates, usage and the species evaluated.^60^, ^61^ Also, herbicide resistance is an important contributing factor to antagonistic interactions in weed control. Addition of glyphosate to dicamba showed an antagonistic interaction for the control of herbicide‐resistant kochia [*Bassia scoparia* (L.) A. J. Scott] due to reduced translocation, but provided effective control of susceptible kochia.^62^ According to Lalonde *et al.*,^63^ the disruption of phloem loading by dicamba applications may affect glyphosate translocation in the plant.^47^ An antagonistic reaction was also observed for the control of glyphosate‐resistant giant ragweed but not glyphosate‐susceptible giant ragweed.^64^ The reduction in barnyardgrass control at the RRS was also consistent with previous research that documented a reduction in glyphosate control of junglerice [*Echinochloa colona* (L.) Link] and other grasses when tank‐mixed with dicamba compared to glyphosate alone.^61^, ^65^ Reduction of glyphosate control of grass species by the addition of dicamba was previously reported for barnyardgrass,^52^, ^66^ johnsongrass [*Sorghum halepense* (L.) Pers.] and wild oat (*Avena fatua L*.).^48^, ^60^, ^61^ Application of glyphosate in mixture with dicamba to 30‐cm barnyardgrass induced a 9% decline in control relative to glyphosate alone.^48^ However, no decrease was observed in biomass reduction of giant foxtail in tank‐mixture in the present study. This is likely due to the fact that antagonistic interactions for grass control depend on the targeted grass species^67^ and weed densities.^68^, ^69^, ^70^

Although not observed in this research to a large extent, a high pH can negatively impact herbicide efficacy^71^, ^72^ and VRAs, especially potassium carbonate, may excessively increase the spray pH thereby potentially negatively impacting efficacy. For example, the efficacy of herbicides from the cyclohexanedione chemical family was affected by the choice of adjuvant and decreased as spray solution pH increased above 7. In contrast, efficacy of aryloxyphenoxypropionate herbicides was not affected by spray solution pH nor by the choice of adjuvants.^73^ According to Devkota and Johnson,^74^ optimum efficacy of glyphosate and dicamba required a carrier water that was low in cation concentration with an acidic pH, revealing a consistent reduction of efficacy for controlling common lambsquarters, common ragweed, horseweed (*Erigeron canadensis* L.), Palmer amaranth, giant ragweed, and pitted morningglory (*Ipomoea lacunosa* L.) when using carrier water at pH 9 compared with pH 4. Similarly, the efficacy of 2,4‐D, premixed 2,4‐D plus glyphosate, and glufosinate was greater at acidic compared with alkaline carrier water pH.^71^, ^72^

Dicamba alone produced the largest droplets which is in alignment with previous research.^75^ The decrease of droplet size in tank‐mixture with glyphosate is also consistent with previous research.^76^ Tank‐mixtures were reported to have the ability to induce a dramatic effect on the droplet spectrum and D_V0.5_.^76^ A reduction of the D_V0.5_ was reported when *S*‐metolachlor was added to dicamba+glufosinate+glyphosate and increased the proportion of driftable fines.^76^ This also is a contributing factor to the label requirement that a drift reduction adjuvant (DRA) must be included with certain dicamba tank‐mixtures; however, the mixture used in this research did not require the addition of a DRA.^77^ Dicamba alone produced droplets of the largest size (D_V0.1_, D_V0.5_, D_V0.9_) with different nozzles compared to a tank‐mixture with other herbicides or even with adjuvants.^75^ Likewise, droplets produced by dicamba alone using air induction nozzles were coarser than those produced by dicamba and glyphosate in tank‐mixture.^78^ By producing finer droplets (D_V0.1_, D_V0.5_, D_V0.9_) and increasing the percentage of driftable fines, the dicamba and glyphosate tank‐mixture increased the physical drift potential of the spray solution.

The slight reduction in droplet size (D_V0.1_ and D_V0.5_) and increase in driftable fines (Table 9), as well as the equal droplet velocity (Table 10) caused by the VRA potassium carbonate could result in increased physical spray drift compared to no VRA or potassium acetate.^79^ Furthermore, the addition of different tank‐mixture partners may change how the VRAs impact droplet dynamics as observed with glyphosate (Fig. 3). Further research should be conducted to evaluate the VRA impact on physical spray drift potential and other application dynamics (*i.e*., spray coverage) from dicamba applied alone and in various tank‐mixtures.

Given the crucial role of droplet size on spray drift, deposition, spray coverage, canopy penetration, and biological efficacy^28^, ^29^, ^30^, ^31^, ^32^ spray solutions with smaller droplet size (glyphosate alone and in tank‐mixture with dicamba) would likely have better coverage, thus improving weed control, excluding antagonistic interactions due to chemical incompatibility. However, weed control efficacy depends on multiple interrelated factors. The bigger values of the relative span for glyphosate alone and in tank‐mixture was an indication of a reduction in the uniformity of droplet size distribution when these solutions were used. Differences in droplet velocity dependent on the herbicide solution being used corroborates previous research in which variations in droplet velocity were observed from spray formulations.^80^ In general, coarser droplets in the present study had higher velocities. The tendency of fine droplets is to rapidly lose initial velocity and reach terminal velocity quicker than coarser droplets.^79^, ^80^ Previous research documented a correlation between size and velocity away from the nozzle^29^ which changes depending on the distance away from the nozzle.^80^ Given the critical role of droplet velocity in spray particle drift,^37^, ^38^ the differential velocities reported in this research might have some impact on weed control. Stopping distances are known to be increased by greater exit velocities while smaller droplets, with lower terminal velocities, resulted in greater leaf adhesion,^31^, ^40^ a greater deposition on vertical plant surfaces, and greater leaf retention.^41^ Future research is needed to fully improve understanding of VRAs impact on droplet dynamics and weed control.

In conclusion, potassium acetate stabilized spray solution pH across herbicides while potassium carbonate increased the pH and had more variability depending on the tank‐mixture. The tank‐mixture of dicamba and glyphosate induced an antagonistic interaction at the RRS location likely due to increased weed densities, the weed species exhibiting higher herbicide tolerances, and reduced uptake of the herbicides in these weed species.^81^ A reduction in spray pH, droplet size, and weed control was observed from mixing dicamba and glyphosate. It may be advisable to avoid tank‐mixtures of these herbicides and instead, apply them sequentially to maximize effectiveness. The VRAs minimally impacted weed control in the present research, but the spray pH increases (particularly by potassium carbonate) may lead to reduced efficacy in certain areas. Additionally, VRAs affected droplet dynamics (droplet size and velocity) differently, which may impact downstream consequences of applications such as coverage, weed control, and physical spray drift.

## CONFLICT OF INTEREST

The authors declare no conflict of interest.

## Data Availability

The data that support the findings of this study are available from the corresponding author upon reasonable request.
